# Skeletal Muscle Mass Measurement Using Cone-Beam Computed Tomography in Patients With Head and Neck Cancer

**DOI:** 10.3389/fonc.2022.902966

**Published:** 2022-06-28

**Authors:** Wei Huang, Peixin Tan, Hongdan Zhang, Zhen Li, Hui Lin, Youxing Wu, Qinwen Du, Qidi Wu, Jun Cheng, Yu Liang, Yi Pan

**Affiliations:** ^1^ Department of Radiation Oncology, Guangdong Provincial People’s Hospital, Guangdong Academy of Medical Sciences, Guangzhou, China; ^2^ Department of Nutrition, Guangdong Provincial People’s Hospital, Guangdong Academy of Medical Sciences, Guangzhou, China; ^3^ National-Regional Key Technology Engineering Laboratory for Medical Ultrasound, Guangdong Key Laboratory for Biomedical Measurements and Ultrasound Imaging, School of Biomedical Engineering, Health Science Center, Shenzhen University, Shenzhen, China; ^4^ Medical Ultrasound Image Computing (MUSIC) Laboratory, Shenzhen University, Shenzhen, China; ^5^ Marshall Laboratory of Biomedical Engineering, Shenzhen University, Shenzhen, China

**Keywords:** malnutrition, chemoradiotherapy, cross sectional, skeletal muscle area, cone-beam computed tomography; computed tomograph, head and neck cancer

## Abstract

**Background and purpose:**

Head and neck cancer (HNC) patients usually present with malnutrition during radiotherapy, leading to loss of skeletal muscle mass (SMM) and poor clinical outcomes. CT has been used in clinical practice for measuring SMM in cancer patients. However, its clinical application for monitoring SMM is limited by the expensive price and high radiation exposure. This study aimed to investigate the feasibility of cone-beam computed tomography (CBCT) for assessing SMM and its changes in HNC patients undergoing radiotherapy.

**Materials and methods:**

This study was divided into two parts. In part 1 (n = 32), the cross-sectional of skeletal muscle area (SMA) at the third cervical vertebra (C3) based on CBCT and computed tomography (CT) was assessed. In part 2 (n = 30), CT and CBCT were performed, and patients’ weight was measured before and at four different time points during radiotherapy. SMAs at C3 were independently identified by three senior radiation oncologists. The interobserver agreement of SMA on CBCT (SMA_CBCT_) findings was analyzed using the intraclass correlation coefficient (ICC). One-way analysis of variance was used to evaluate the interobserver variability and statistical significance for SMA measurements. CBCT and CT measurement differences and correlations were analyzed using paired sample *t*-test and Pearson correlation analysis, respectively. The Krouwer variant of the Bland–Altman plot was used to analyze the agreement of SMA measurements between CBCT and CT. A simple linear regression model was used to analyze the relationship of SMA measurements between the two imaging techniques, and the equation was established. A repeated-measures ANOVA was performed to evaluate the effects and interactions between weight loss, SMA loss, and time.

**Results:**

SMA_CBCT_ demonstrated excellent interobserver reliability; no significant difference between SMA_CBCT_ and SMA on CT (SMA_CT_) at C3 was observed in all patients. The SMA_CBCT_ and SMA_CT_ were highly correlated (*r* = 0.966; 95% confidence interval = 0.955–0.975; *p* < 0.001). Bland–Altman analysis revealed that SMA_CBCT_ was generally higher than SMA_CT_. The predicted SMA value at C3 on CT using CBCT was similar to the actual value. Moreover, significant differences between SMA and weight loss (*F* =10.99, *p* = 0.002), groups (weight loss and SMA loss) and times (4 time points) (*F* = 3.93, *p* = 0.013), and mean percent loss over time (*F* = 7.618, *p* < 0.001) were noted.

**Conclusion:**

CBCT may be used as an alternative for CT to measure SMA in HNC patients during radiotherapy.

## Introduction

Chemoradiotherapy is the standard treatment for unresectable locally advanced head and neck cancer (HNC). However, patients undergoing radiotherapy experience adverse effects, such as mucositis, dysgeusia, nausea, and vomiting, leading to inadequate food intake and weight loss, ultimately resulting in malnutrition. During chemoradiotherapy, 44–88% of HNC patients present with malnutrition (1), thereby contributing to the loss of skeletal muscle mass (SMM) and function associated with adverse clinical outcomes, including treatment interruption (2), infection, longer hospital stays (3), and poor survival rates (4, 5). The Global Leadership Initiative on Malnutrition recommends the inclusion of low muscle mass in the diagnostic criteria for malnutrition (6). To effectively manage HNC patients, timely SMM assessment and early malnutrition intervention are important during radiotherapy. However, body composition and skeletal muscle loss are not accurately reflected by the currently available tools for assessing malnutrition, which only measure body mass index (BMI) and weight loss. Therefore, the accurate assessment of SMM and its changes during radiotherapy remains a challenge.

Currently, different assessment tools are used to measure SMM, including anthropometry, dual-energy X-ray absorptiometry, bioelectrical impedance analysis, computerized tomography (CT), and ultrasonography. However, no consensus has been established on the best technique for muscle mass measurement, as all of them are indirect measurement methods. CT has been used in clinical practice for measuring SMM in cancer patients by calculating the cross-sectional skeletal muscle areas (SMAs) at the third lumbar vertebra (L3); this area is highly correlated with total body muscle mass and provides precise and quantitative information (7, 8). In addition to the L3, the cross-sectional SMA at the third cervical vertebra (C3) has been recently shown to be a reliable and accurate surrogate in HNC patients (9). However, the clinical application of these methods for monitoring SMM is limited by the expensive price and high radiation exposure.

Cone-beam CT (CBCT), a variation of traditional CT, has been widely used in clinical settings to provide three-dimensional images for diagnosis and imaging guidance. CBCT as an effective image-guided radiotherapy tool is widely performed during radiotherapy and produces CT images of the target region to ensure the appropriate position of the patients. To the best of our knowledge, its use for assessing SMM changes has not previously been investigated. We hypothesized that the cross-sectional SMA at C3 on CBCT would be a reliable alternative to SMA at C3 on CT in patients undergoing radiotherapy. This study aimed to assess the feasibility and reliability of measuring SMM and its changes using CBCT in HNC patients undergoing radiotherapy.

## Materials and Methods

### Patients and Study Design

Patients with locally advanced HNC scheduled to receive radical radiotherapy with the following characteristics were prospectively enrolled in this study: age ≥ 18 years, histologically confirmed HNC, clinical stage II–IVa according to the American Joint Committee of Cancer (7th edition), and Eastern Cooperative Oncology Group (ECOG) performance status 0–1. Exclusion criteria were previous head and neck radiotherapy or cervical lymph node dissection, active infections, palliative treatment, and incomplete range scan.

This study was divided into two parts. In the first part, CT and CBCT were performed in 32 HNC patients before radiotherapy at the 0th fraction, 15th, and 25th fractions. If a correlation was observed between the SMA at C3 on CBCT (SMA_CBCT_) and CT (SMA_CT_) (Pearson correlation coefficient ≥0.7), new patient enrolment for part 2 with the same eligibility and exclusion criteria of part 1 was then opened. The second part was a longitudinal study wherein CT, CBCT, and body weight data were obtained on the 0th fraction, followed by CBCT scan and consecutive body weight measurements during radiotherapy (5th, 10th, 15th, and 25th fractions). SMAs at C3 on 0th fraction scans were identified by three independent senior radiation oncologists (observers 1, 2, and 3 with > 10-year experience in radiation oncology); observer 1 contoured the remaining fractions.

The study was conducted in compliance with local and national regulations and was approved by the Ethics Committee of the Guangdong Provincial People’s Hospital (approval no. GDREC2018296H(R1)). Written informed consent was obtained from all patients.

### Image Acquisition and Skeletal Muscle Measurements

All patients were immobilized with a thermoplastic head-and-shoulder mask and underwent CT and CBCT scans. Neck CT was performed (tube voltage: 120 kVp; slice thickness: 2.5 mm; matrix: 512 × 512) in accordance with department standard procedures. The CBCT images were acquired using the Varian CBCT (Trilogy; American; tube voltage: 100 kVp; slice thickness: 2.5 mm; matrix: 384 × 384). A 25-cm field of view (FOV) was used in the full-fan mode.

**Figure 1:**
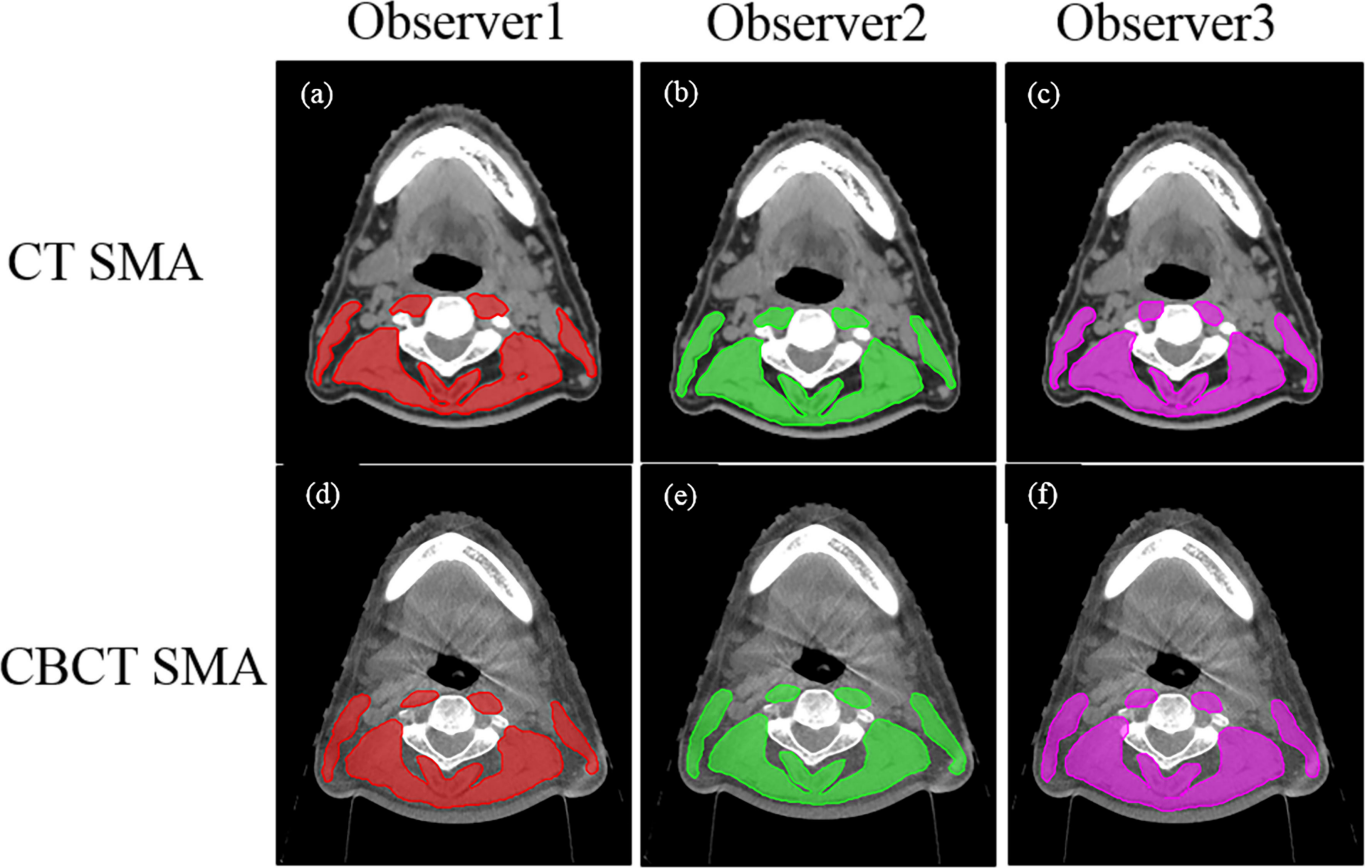
Computed tomography (CT) and cone-beam CT (CBCT) images identified by three independent senior radiation oncologists (observers 1, 2, 3). The upper panel **(A–C)** shows the CT skeletal muscle mass (29–150 HU) and the lower panel **(D–F)** shows the CBCT skeletal muscle mass (observer 1, red; observer 2, green; observer 3, pink).

During CT imaging, skeletal muscle segmentation and SMM quantification were performed according to the established method published by Swartz et al. (9) (Hounsfield unit [HU] ranges, −29 +150 HU). The SMA of the CBCT image was identified using HU thresholds of −300+1800 HU. The SMAs on both CT and CBCT images were identified by three independent senior radiation oncologists (observers 1, 2, and 3) (**Figure 1**). Furthermore, the protocol for single-slice selection and SMA was predefined. As previously described (9), the first slice was obtained while scrolling through C3 in a caudo-cephalic direction to demonstrate both transverse processes and the vertebral arch completely. The cross-sectional area of the SMA at C3 was measured by a physicist using the Matlab software (version 2019b) by summing the area of the selected muscle pixels. The radiation oncologist were blinded to the measurements when delineating SMAs.

### Statistical Analysis

Continuous data are presented as the mean ± standard deviation (SD), and categorical data as numbers and percentages of the total. The interobserver agreement of SMA_CBCT_ findings was analyzed using the intraclass correlation coefficient (ICC) with 95% confidence intervals (CI) based on a mean rating (κ = 3), absolute-agreement, and two-way random model. ICC values <0.5 indicate poor agreement, values between 0.5 and 0.75 indicate moderate agreement, values between 0.75 and 0.9 indicate good agreement, and values >0.90 indicate excellent agreement (10). One-way analysis of variance (ANOVA) was used to evaluate the interobserver variability and SMA measurements. CBCT and CT measurement differences and correlations were analyzed using paired sample *t*-test and Pearson correlation analysis, respectively. The Krouwer variant of the Bland–Altman plot was used to analyze the agreement of SMA measurements between CBCT and CT scans. For each comparison, the 95% limits of agreement were computed as the average difference ±1.96 SD of the difference. A simple linear regression model was used to analyze the relationship of SMA measurements at C3 between the two imaging techniques, and an equation was established to predict SMA_CT_ value at C3 from SMA_CBCT_ value. CBCT and CT performed at the 15th and 25th fractions of radiotherapy in the first part were used to validate the model. The residual values between the predicted values and the actual values were calculated, and the agreement was assessed using the Bland–Altman plot. A repeated-measures ANOVA was performed to evaluate the effects and interactions among weight loss, SMA loss, and time. All statistical analyses were performed using the IBM SPSS Statistics package version 23 (SPSS Inc., Illinois, USA). Statistical significance was set at *P* < 0.05.

## Results

**Figure 2:**
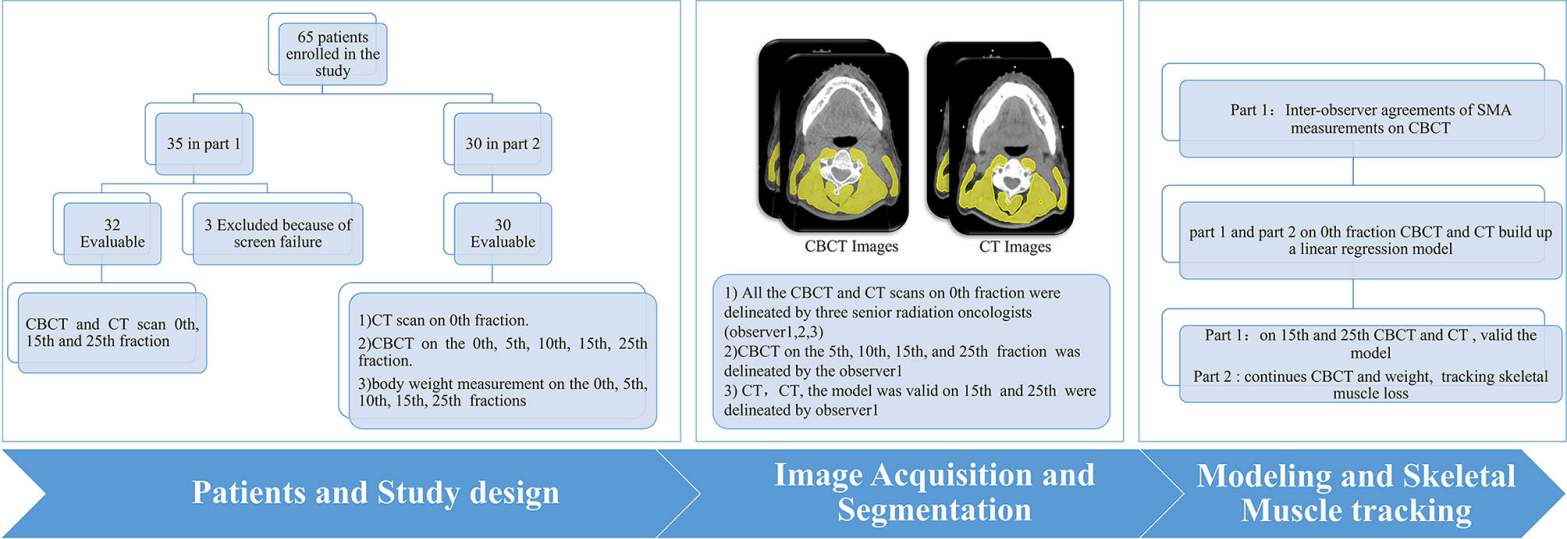
Workflow scheme.

Between October 2019 and September 2020, 65 HNC patients were enrolled in the study, 35 in part 1 and 30 in part 2 (**Figure 2**). In part 1, three patients were excluded because the entirety of their muscles at the C3 level was not covered by the region of interest of the CBCT scan (**Figure S1**). Patient characteristics are shown in **Table 1**.

**Table 1:** Patient characteristics (N=62).

Characteristics	Total (N=62)	Part 1 (n=32)	Part 2 (n=30)
Sex			
Male	42 (67.7%)	22 (68.8%)	20 (66.7%)
Female	20 (32.3%)	10 (31.3%)	10(33.3%)
Age, years, mean ± SD (range)	49.95 ± 11.22 (23–83)	50.84 ± 12.5 (23–83)	49 ± 9.7 (28–67)
Height, cm	164.177 ± 7.57	165.09 ± 6.94	163.2 ± 8.19
Weight, kg	63.35 ± 12.73	61.98 ± 15.49	64.8 ± 8.9
Site of malignancy			
Nasopharyngeal	42 (67.7%)	12 (37.5%)	30 (100%)
Parotid	7 (11.3%)	7 (21.9%)	0
Oral cavity	7 (11.3%)	7 (21.9%)	0
Hypopharyngeal	3 (4.8%)	3 (9.4%)	0
Others	3 (4.8%)	3 (9.4%)	0
T stage			
T1	2 (3.2%)	2 (6.3%)	0
T2	27 (43.5%)	12 (37.5%)	15 (50%)
T3	24 (38.7%)	11 (34.4%)	13 (43.3%)
T4	9 (14.5%)	7 (21.9%)	2 (6.7%)
N stage			
N0	6 (9.7%)	5 (15.6%)	1 (3.3%)
N1	21 (33.9%)	8 (25%)	13 (43.3%)
N2	31 (50%)	15 (46.9%)	16 (53.3%)
N3	4 (6.5%)	4 (12.5%)	0
Radiation dose, Gy, (range)	68.6 ± 17.6 (60–71)	68.2 ± 19.6 (60–71)	69.1 ± 13.9 (68–71)
Chemoradiotherapy			
Radiotherapy alone	8 (12.9%)	8 (25%)	0
Sequential	3 (4.8%)	3 (9.4%)	0
Concurrent	51 (82.3%)	21 (65.5%)	30 (100%)

The SMA at C3 measurements at the 0th fraction identified by the three observers are shown in **Table 2**; no significant difference was found among the SMA at C3 from the CBCT or CT scan of the 62 patients. The ICC values were excellent (all >0.95; *p* < 0.001; **Table 2**).

**Table 2:** SMA measurement at the level of C3 on CT and CBCT.

		SMA, cm^2^			ICC values (95% CI)	*P*	*P* ^a^
		Obs. 1	Obs. 2	Obs. 3			
CBCT	Part 1	34.16 ± 8.4	34.99 ± 8.6	34.28 ± 8.8	0.985 (0.971–0.992)	<0.001	0.917
	Part 2	34.98 ± 7.9	36.81 ± 8.4	35.41 ± 8.1	0.973 (0.899–0.990)	<0.001	0.665
	Total	34.56 ± 8.2	35.87 ± 8.4	34.82 ± 8.4	0.979 (0.952–0.989)	<0.001	0.653
CT	Part 1	32.54 ± 7.86	33.82 ± 8.35	31.83 ± 8.52	0.958 (0.894–0.981)	<0.001	0.621
	Part 2	32.85 ± 8.0	34.09 ± 8.7	32.89 ± 7.32	0.954 (0.912–0.977)	<0.001	0.973
	Total	32.69 ± 7.8	33.95 ± 8.4	32.34 ± 7.9	0.955 (0.916–0.975)	<0.001	0.506

CBCT, cone-beam computed tomography; CI, confidence interval; CT, computed tomography; obs., observer; SME, skeletal muscle area.

a P values analyzed by one-way analysis of variance.

**Figure 3:**
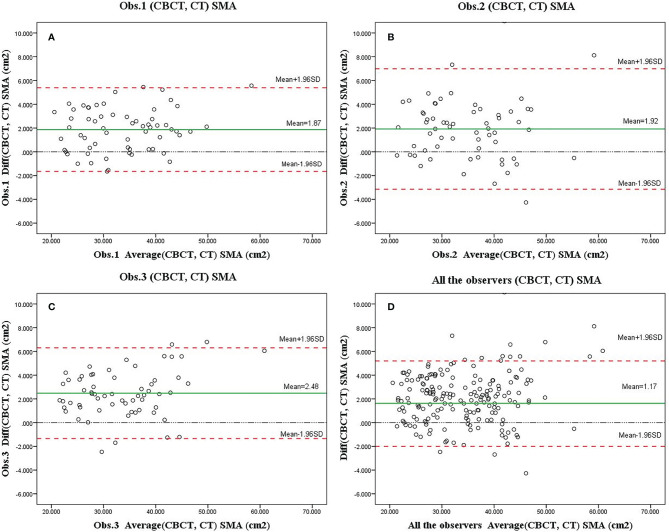
Bland–Altman plots of skeletal muscle area (SMA) measurements at the level of C3 based on the cone-beam computed tomography (CBCT) and computed tomography (CT) scans as assessed by three observers **(A–C**, showed separately; **D**, showed altogether). Systematic bias estimates (mean, solid red line, and 95% limits of agreement) (mean difference ±1.96 standard deviation of the difference) are shown.

The mean SMA at C3 at 0th fraction of all patients was 34.64± cm^2^ and 32.6 ± cm^2^ on CBCT and CT, respectively. The SMA measurement at C3 on CBCT was highly correlated with the CT measurement (*r* = 0.966, 95% CI = 0.955–0.975; *p* < 0.001). A significant difference was found among the SMA measurements from the CBCT and CT scans identified by the three observers (observer 1: 34.56 ± 8.19 vs. 32.69 ± 7.87, *P* < 0.001; observer 2: 34.83 ± 8.43 vs. 32.34 ± 7.92, *P* < 0.001; observer 3: 35.88 ± 8.50 vs. 33.59 ± 8.43, *P* < 0.001). The Bland–Altman plots are shown in **Figure 3**; the mean difference and 95% limits of agreement among observers 1, 2, and 3 were as follows: 1.65 with 5.39, −3.15 with 6.99, and −1.34 with 6.31. There was a systemic bias in the SMA measurements at C3 between CBCT and CT scans (observer 1 bias = 1.86; observer 2 bias = 1.92; observer 3 bias = 2.48) (*p* < 0.001), which indicates larger SMA measurements on the latter. The actual difference among observers was 2.09 ± 2.14 cm^2^ (range: −4.26 to 10.99).

**Figure 4:**
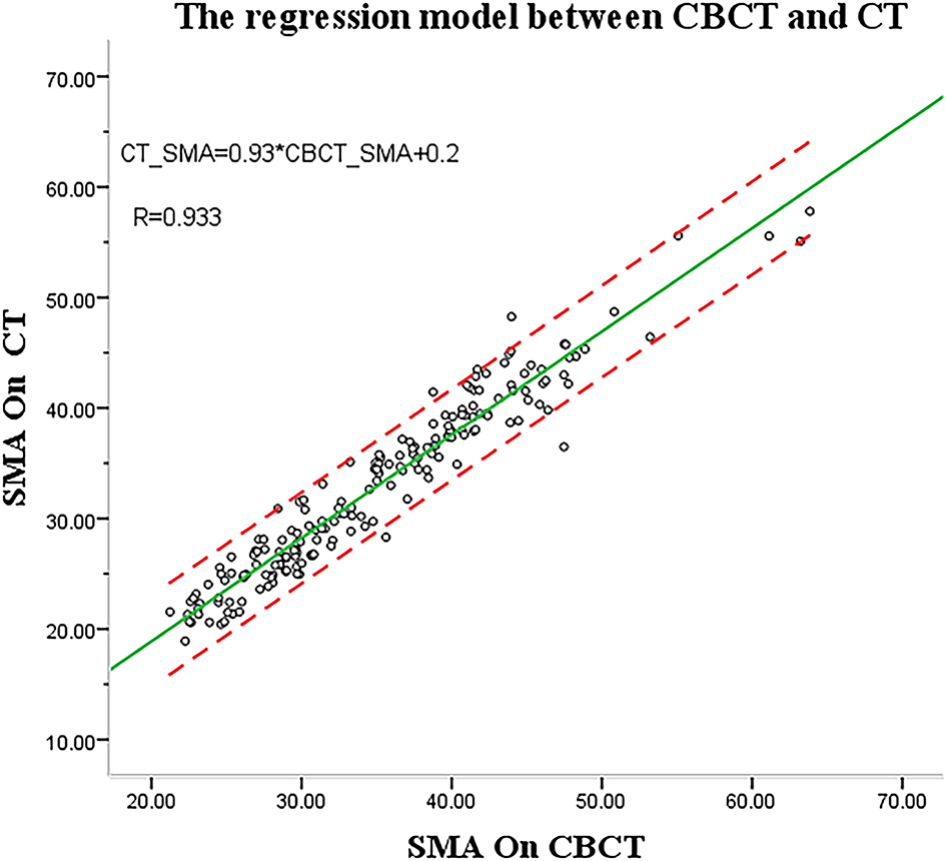
Analysis of skeletal muscle estimation using cone-beam computed tomography (CBCT).

In part 1, the regression equation was SMA_CT_ = y = 0.2 (−0.46; 0.86) + 0.93 (0.92; 0.94) SMA_CBCT_ (**Figure 4**). The CBCT and CT imaging performed at the 15th and 25th fractions of radiotherapy in part 1 were used as test data to validate the linear regression model of SMA at C3, CBCT, and CT. SMA_CT_ was estimated based on the SMA_CBCT_ and compared with the actual value.

**Figure 5:**
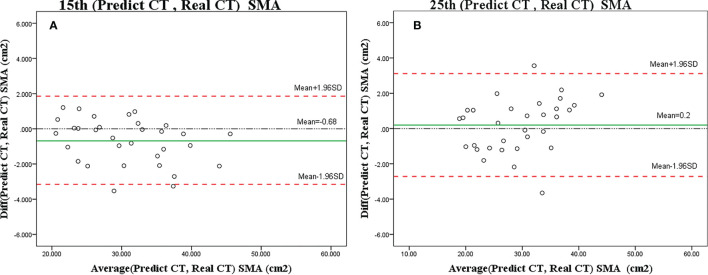
Bland–Altman plots showing the agreement between predicted SMA and actual SMA based on the CT scan on the 15th **(A)** or 25th **(B)** fraction. The difference (diff) between measurements is shown on the y-axis, and the average value of the two measurements is shown on the x-axis.

Only 2 (6%) patients were beyond the 95% limits of agreement (95% LOA) between measurements, implying a reasonable agreement. The predicted SMA value at C3 using CBCT was similar to the actual value (15th, *r* = 0.984, *p* < 0.001; 25th, *r* = 0.978, *p* < 0.001) (**Figures 5A, B**), with no significant difference (ANOVA: 15th, *p* = 0.690; 25th, *p* = 0.907).

**Figure 6:**
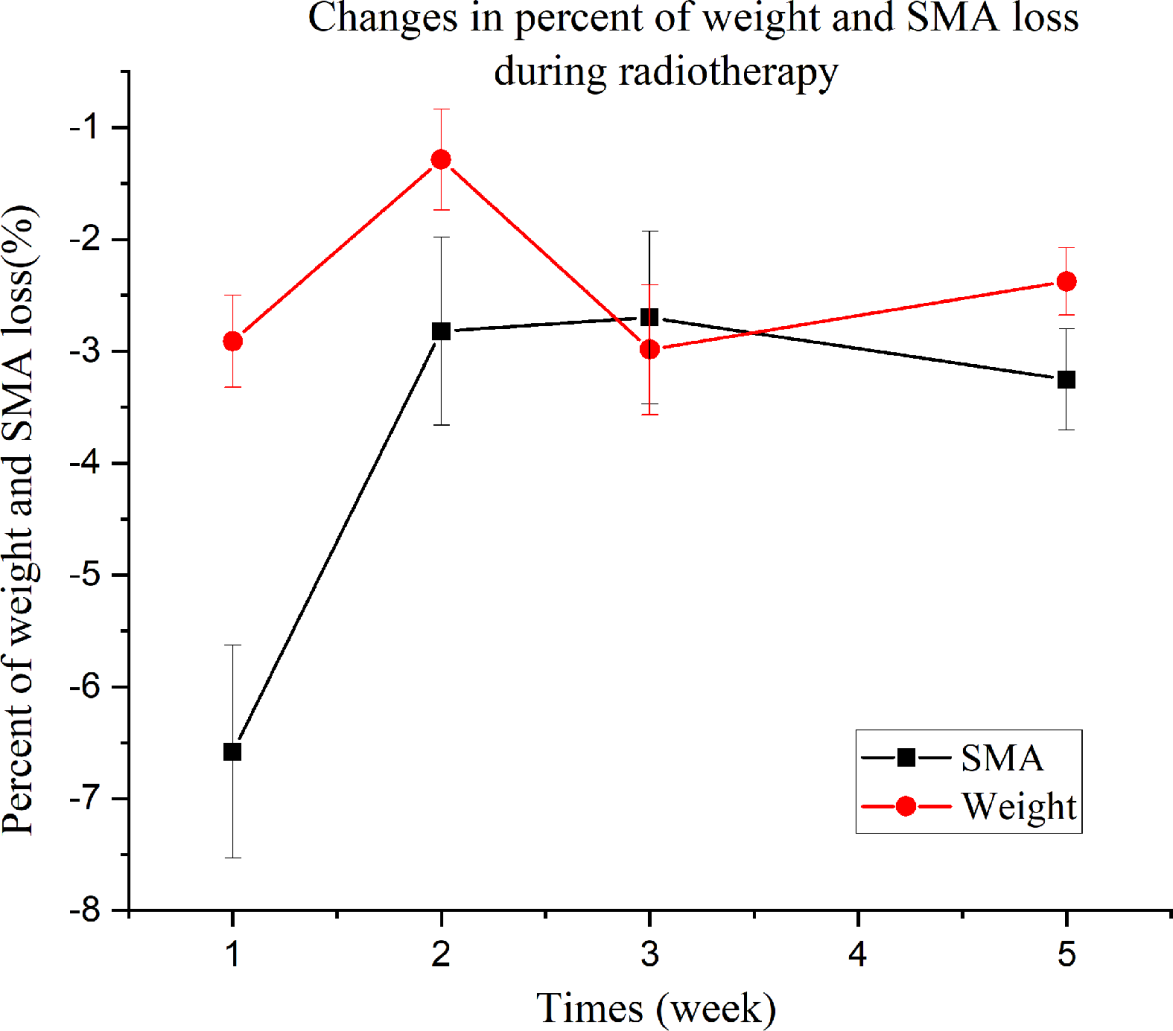
Percentage changes in weight and skeletal muscle area loss during radiotherapy.

The results of the repeated measures ANOVA are shown in **Figure 6**. Weight and SMA loss significantly increased over time, with a highly significant difference between the mean percentages of SMA at C3 and weight loss (*F* = 10.99 [df=1], *p* = 0.002). Additionally, a highly significant difference in the percent loss over time was observed (*F* = 7.618 [df = 2.591], *p* < 0.001). The test for the interaction between group (weight loss and SMA loss) and time was significant (*F* = 3.93 [df = 2.591], *p* = 0.013). Percent weight loss increased in the first and last two weeks of radiotherapy, whereas percent SMA loss rapidly increased during the first week. The magnitude of increase in mean percent SMA loss and mean percent weight loss in week 1 was 6.6% and 2.9%, respectively.

## Discussion

The measurement of SMM can identify the nutritional risk and monitor the progress on malnutrition in HNC patients (11–13). The CT-based technique is a reference objective method for skeletal muscle measurement, as the SMA has a high correlation with whole-body mass (14–16). However, it is usually performed as a diagnostic or evaluative tool for patients before or after cancer treatment and is not regularly done during radiation therapy. Compared with CT, CBCT requires a lower radiation dose with 3–5 times less cost; however, CBCT is limited by its soft tissue imaging quality. To our knowledge, this is the first study to investigate the feasibility of CBCT for assessing SMM and its changes in HNC patients during radiotherapy.

Our findings showed that the interobserver reliability was excellent for SMA at the level of C3 on both CBCT and CT scans. Thus, the results indicate the reproducibility and reliability of CBCT between multiple observers. Fourie et al. (17) investigated the accuracy of CBCT to measure the soft tissue thicknesses of the face in cadavers and similarly observed an excellent interobserver agreement. However, using CBCT, Giovanni et al. (18) reported mean ICC values for soft tissue thickness of 0.49 and 0.66 in patients with facial linear scleroderma and age-matched healthy controls, respectively, which was lower compared in our study. We attribute this discrepancy to the difference in measurement location. In the previous study, ICC was higher at the level of mandibular foramen and lower at the maxillary sinus, whereas ICCs were >0.98 at the C3 level in our study. Similar to our study, Zwart et al. (12) and Bril et al. (19) reported a excellent ICC value for SMA at C3 on CT, respectly. Compared to CBCT, CT may has the better interobserver ICC for neck muscles. It is likely that the interobeserver agreement is lower when the image resolution is lower.

SMA is usually measured on CT; therefore, evaluating the correlation between CBCT and CT is important to establish CBCT as an effective skeletal muscle assessment method. We found a high correlation between SMA measurements on CBCT and CT at the C3 level and built a model to estimate SMA_CT_ with reference to SMA_CBCT_ at the same time point. The estimated SMA value at C3 was close to the actual value on CT, and there was no significant difference between the predicted and the actual values. However, a significant difference was observed between the SMA measurements of CBCT and CT. The Bland–Altman analysis demonstrated a 2% bias between the two modalities, indicating that CBCT may overestimate the SMA. This may be due to the lower soft tissue imaging resolution of CBCT compared with CT. Furthermore, CBCT contains smaller flat-panel detectors, which receive lower photon flux, resulting in inferior image quality than CT (20, 21). The voxel sizes influence spatial resolution of CBCT which is an important parameter to image quality. The selection of voxel size is useful to soft tissue measurement on CBCT (22). A specific protocol needs to be defined for SMA measurement at C3 on CBCT.

To investigate the effectiveness of CBCT as a tool for monitoring SMM during radiotherapy, we conducted a longitudinal study with consecutive measurements of body weight and SMA using CBCT. The results showed that patients during radiotherapy continued to lose SMA with a mean percentage of 16%. However, non-uniform SMA loss was observed during treatment. In particular, a rapid percent SMA loss was observed in the first week, whereas percent weight loss was not noticeable, indicating a significant difference between percent SMA and weight loss. Compared with skeletal muscle loss, weight loss is a well-validated but inaccurate indicator of nutritional status and by itself cannot predict outcomes (23). Our results showed that weight loss did not reflect SMA loss in patients undergoing radiotherapy. HNC patients experiencing SMA loss during chemoradiotherapy have been recently reported to have worse overall survival and poorer quality of life (23–25).

However, our study has several limitations. First, three patients were excluded because their CBCT scans were off-center; therefore, the skeletal muscle at the C3 level in these patients extended outside the FOV. A full-fan cone beam with an FOV of 240 mm was used according to the head and neck protocol in the Varian OBI system; additionally, the scanner and radiation centers were aligned with each other. However, during ipsilateral irradiation, the radiation center deviated far from the center of the body; therefore, the region of interest of the scan did not cover the whole neck at an axial plane owing to the small FOV. Hence, the CBCT scanner center should be placed at the center of the body when measuring SMA by CBCT. Second, this pilot study utilized a relatively small sample size, particularly in the longitudinal study (Part 2). A phase II study is currently being designed to investigate the value of CBCT in monitoring SMA loss during radiotherapy.

Overall, this study demonstrated that measuring SMA at C3 and its changes using CBCT during radiotherapy is feasible and shows excellent reliability and reproducibility among multiple raters. Although it may overestimate SMA, CBCT during radiotherapy remains a reliable alternative as demonstrated by good agreement, linear correlation, and linear regression models constructed based on SMA measurements obtained from CBCT and CT at the level of C3. Additionally, a non-uniform pattern of SMA loss was found during radiotherapy, indicating that CBCT has the potential to monitor changes in SMA during radiotherapy.

## Data Availability Statement

The raw data supporting the conclusions of this article will be made available by the authors, without undue reservation.

## Ethics Statement

The studies involving human participants were reviewed and approved by the Ethics Committee of Guangdong Provincial People’s Hospital (approval no. 2018-296H-2). The patients/participants provided their written informed consent to participate in this study

## Author Contributions

WH and YP designed and directed the project. HZ, ZL, HL, YW, QD and QW were responsible for the treatment and clinical care of the patients. PT, JC and YL collected the data. YP and WH contributed to manuscript writing, critical review and editing of the paper. All authors have read and approved the final manuscript.

## Funding

This work was funded by The Medical Scientific Research Foundation of Guangdong Provincial People’s Hospital (grant number 2018lcpx09), the Wu JiePing Medical Foundation (grant number 320.6750.19089-1), the National Natural Science Foundation of China (grant number 61901275), the Natural Science Foundation of SZU (grant number 2019131), the Guangzhou Science and Technology Plan Foundation (2021-02-01-04-1002-0017) and the Guangdong Medical Research Foundation (grant number A2019260) funded the study.

## Conflict of Interest

The authors declare that the research was conducted in the absence of any commercial or financial relationships that could be construed as a potential conflict of interest.

## Publisher’s Note

All claims expressed in this article are solely those of the authors and do not necessarily represent those of their affiliated organizations, or those of the publisher, the editors and the reviewers. Any product that may be evaluated in this article, or claim that may be made by its manufacturer, is not guaranteed or endorsed by the publisher.

## Abbreviations

CBCT, cone-beam computed tomography; CT, computed tomography; HNC, head and neck cancer; SMA, skeletal muscle area; SMM, skeletal muscle mass.
