# Supplementary figures and images for: Skeletal Muscle Mass Measurement Using Cone-Beam Computed Tomography in Patients With Head and Neck Cancer
Source: Front Oncol. 2022 Jun 28;12:902966. doi: 10.3389/fonc.2022.902966 (PMC9273748; doi:10.3389/fonc.2022.902966)

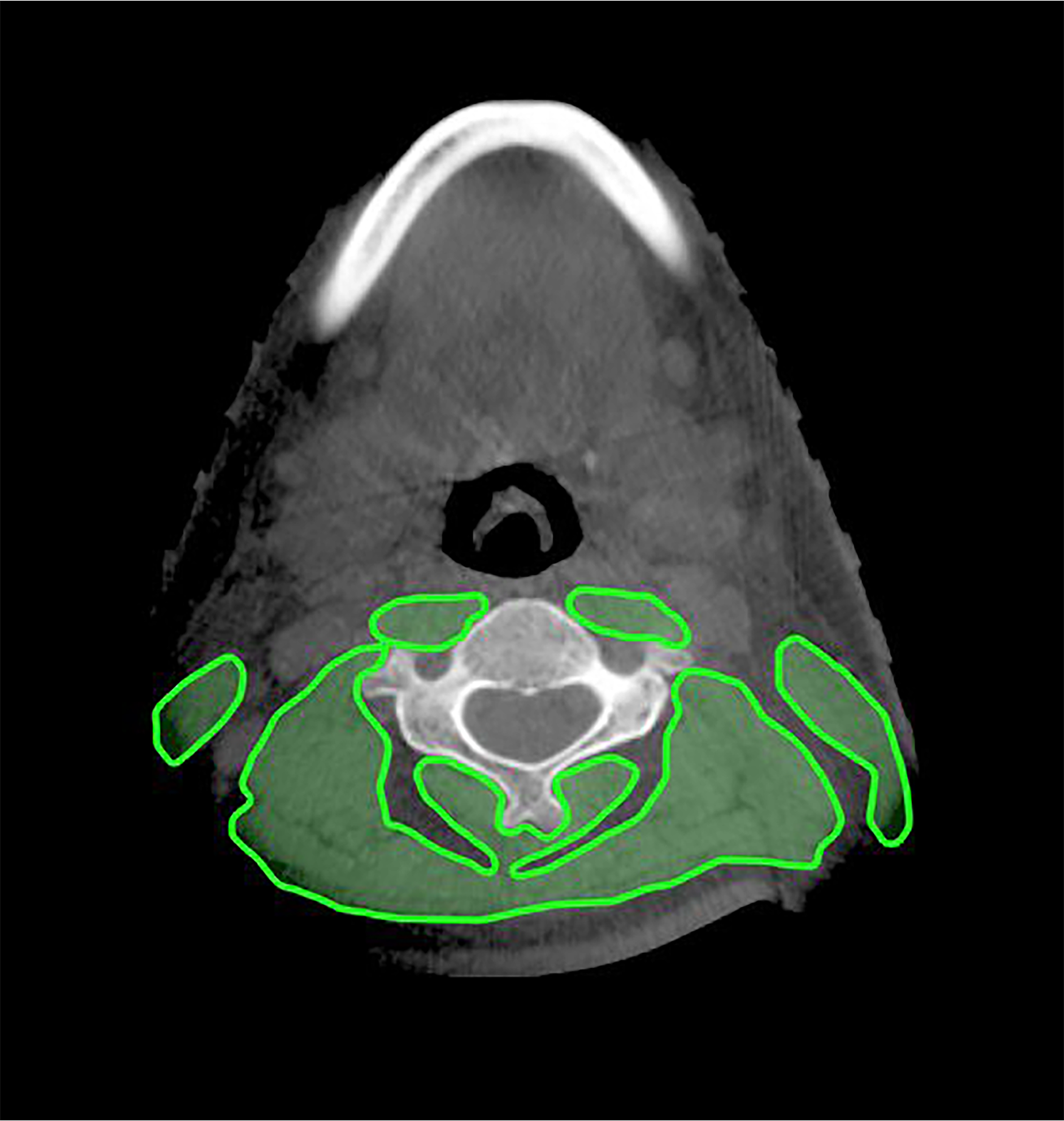

Supplement: Supplementary file 1 [file Image_1.tif]
